# Rapid emergence of dolutegravir resistance on second-line dolutegravir-based ART

**DOI:** 10.4102/sajhivmed.v26i1.1701

**Published:** 2025-04-23

**Authors:** Kirusha Naidoo, Richard J. Lessells, Jienchi Dorward, Mahomed Y.S. Moosa, Yukteshwar Sookrajh, Pravi Moodley, Paul K. Drain, Nigel Garrett

**Affiliations:** 1 Department of HIV Vaccines and Pathogenesis, Centre for the AIDS Programme of Research in South Africa (CAPRISA), Durban, South Africa; 2 KwaZulu-Natal Research Innovation and Sequencing Platform (KRISP), College of Health Sciences, University of KwaZulu-Natal, Durban, South Africa; 3 Nuffield Department of Primary Care Health Sciences, University of Oxford, Oxford, United Kingdom; 4 Department of Infectious Diseases, Nelson R. Mandela School of Medicine, University of KwaZulu-Natal, Durban, South Africa; 5 eThekwini Municipality Health Unit, Durban, South Africa; 6 Department of Virology, School of Laboratory Medicine and Medical Sciences, University of KwaZulu-Natal, Durban, South Africa; 7 Department of Virology, National Health Laboratory Service, Inkosi Albert Luthuli Central Hospital, Durban, South Africa; 8 Department of Epidemiology, University of Washington, Seattle, Washington, United States of America; 9 Department of Global Health, University of Washington, Seattle, Washington, United States of America; 10 Department of Medicine, University of Washington, Seattle, Washington, United States of America; 11 Department of Public Health Medicine, School of Nursing and Public Health, University of KwaZulu-Natal, Durban, South Africa

**Keywords:** drug resistance, dolutegravir resistance, drug-drug interaction, dolutegravir, rifapentine

## Abstract

The integrase strand transfer inhibitor, dolutegravir (DTG), is widely used in first- and second-line antiretroviral therapy (ART) regimens in South Africa. We describe an adult with virological failure on second-line tenofovir/lamivudine/dolutegravir (TLD2) and rapid emergence of DTG resistance within 3 months, while receiving rifapentine-based tuberculosis preventive therapy.

**What this study adds:** This case demonstrates rapidly emerging DTG resistance and explores the interaction between rifapentine and DTG as a likely contributor to virological failure. This highlights the need for HIV guidelines to allow for early genotype resistance testing in viraemic individuals where drug-drug interactions and other risk factors for resistance are identified.

## Introduction

The South African National Department of Health (SANDoH) adopted the use of dolutegravir (DTG)-based antiretroviral therapy (ART) in 2019,^1^ because of its efficacy and high genetic barrier to resistance.^2,3^ Since then, DTG resistance has gradually been emerging in clinical settings, raising concerns regarding the sustainability of current ART regimens.

Emergent DTG resistance is more common among ART-experienced individuals who transition from non-nucleoside reverse transcriptase inhibitor (NNRTI)-based ART to a DTG-containing regimen while viraemic.^3^ Other risk factors include DTG mono- or dual-therapy or reduced drug concentrations.^3^ SANDoH guidelines reserve HIV drug resistance testing for patients with sustained viraemia ≥ 1000 copies/mL following two or more years on a DTG-based ART regimen, with adequate adherence.^2^

## Ethical considerations

Ethical clearance to conduct this study was obtained from the University of KwaZulu-Natal Biomedical Research Ethics Committee (reference no.: BRECBREC/00000833/2019), who provided full ethical approval for the study on 11 February 2020. Yearly recertification has been obtained. Written informed consent was obtained from the participant.

## Patient presentation

A 36-year-old male was enrolled in the Simplifying Treatment and Monitoring for HIV (STREAM HIV) phase IIb randomised control trial to evaluate the impact of point-of-care (POC) urine tenofovir adherence and POC viral load (VL) testing on treatment adherence in South Africa.^4^ On 12 December 2022, he was diagnosed with WHO stage three HIV infection, with a baseline CD4+ T-cell count of 123 cells/µL and rifampicin-sensitive pulmonary tuberculosis (TB). He was initiated onto TB therapy and co-trimoxazole prophylaxis, and ART was deferred as per national guidelines.^1^ After 4 weeks of TB therapy, he was enrolled into the clinical trial and was randomised to the POC testing arm, which allowed for same-day review of POC VL results, use of urine tenofovir assays to confirm adherence in cases of viral non-suppression, and real-time genotypic resistance testing, if VL ≥ 200 copies/mL, and tenofovir detectable in urine. Considering the TB coinfection, he was initiated on tenofovir, emtricitabine and efavirenz (TEE) because of its compatibility and efficacy when administered with TB therapy.

After completing a standard 6-month course of TB therapy and achieving clinical and bacteriological cure in June 2023, 3 months of weekly isoniazid and rifapentine (3HP) TB preventive therapy was commenced for secondary prophylaxis.

**FIGURE 1:**
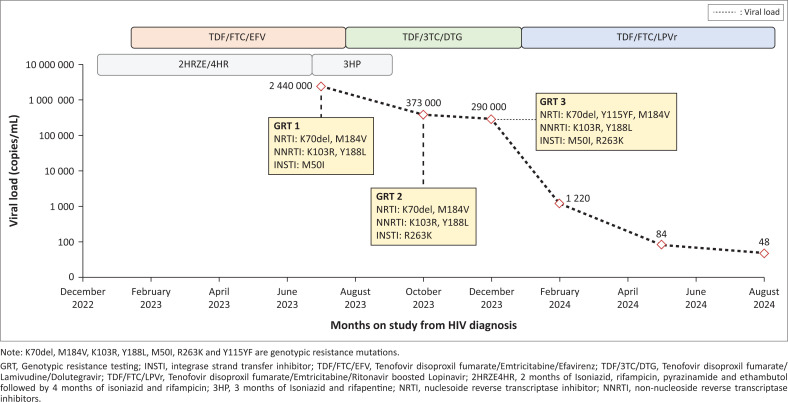
Virological response and resistance mutations over time in relation to antiretroviral therapy and tuberculosis preventive therapy.

An initial VL performed in July 2023, after 6 months on ART, was 2440000 copies/mL (Figure 1). Good adherence was confirmed by self-report, adherence to all scheduled visits and pill refill dates, and a positive POC urine tenofovir test. Genotypic resistance testing revealed the K70del and M184V NRTI mutations, and the K103R and Y188L NNRTI mutations. It also showed the polymorphic M50I integrase mutation. Following updated guidelines recommending DTG for second-line ART,^1^ his regimen was switched to tenofovir, lamivudine and dolutegravir (TLD2) on 20 July 2023, while continuing 3HP. Retrospective pre-ART genotyping detected the Y188L mutation. No major or accessory integrase strand transfer inhibitor (INSTI) mutations were detected.

Despite documented good adherence, he remained viraemic at 3 and 6 months of TLD2, with VLs of 373000 copies/mL at 3 months, and 290000 copies/mL at 6 months. Genotyping after 3 months of TLD2 revealed the R263K major INSTI mutation. No further mutations emerged after 6 months. A protease inhibitor (PI)-based regimen of tenofovir, emtricitabine, and ritonavir-boosted lopinavir was initiated on 10 January 2024 following review of the above results. After 1 month, his VL dropped to 1220 copies/mL and by 7 months, viral suppression of < 50 copies/mL was achieved.

Despite virological failure and the development of DTG resistance, his CD4+ T-cell count after 12 months on ART improved to 241 cells/µL, and co-trimoxazole prophylaxis was discontinued. The participant remained clinically well on follow-up visits, apart from a folliculitis and persistent fungal dermatitis, which resolved a month after switching to a PI-based ART regimen.

## Discussion

This case demonstrates the rapid development of virological failure on an NNRTI-based regimen, and then primary virological failure of TLD2 with the rapid emergence of DTG resistance after only 3 months of DTG exposure. It stresses the need for heightened surveillance and flexibility to ensure early access to genotypic resistance testing in certain clinical situations, including cases of viraemia when drug-drug interactions are suspected.

Virological failure and emergent INSTI resistance have been observed in people living with HIV (PLWH) on second-line DTG-containing ART following virological failure on a NNRTI regimen.^3^ The Y188L mutation present at baseline would have conferred high-level resistance to efavirenz,^5^ facilitating early virological failure on TEE. The NRTI drug resistance mutations present at TEE failure (K70del, M184V) likely emerged under drug pressure and would have conferred lamivudine (3TC) and emtricitabine (FTC) resistance, but would not be expected to affect susceptibility to tenofovir disoproxil fumarate (TDF).^5^ Furthermore, the lack of INSTI resistance mutations at baseline refutes the likelihood of transmitted resistance and the M50I polymorphism detected pre-DTG initiation is known to occur in ~30% of INSTI-naïve individuals with subtype C, and would not be expected to affect susceptibility to DTG in isolation.^5^ Both DTG and TDF would have been expected to be fully active in this case.

The drug-drug interaction between DTG and rifapentine likely contributed to the rapid emergence of DTG resistance, although we do not have DTG drug concentration data. WHO and SANDoH guidelines recommend that 3HP be restricted to those with viral suppression on DTG-based ART.^1,6^ Rifapentine is an inducer of the uridine diphosphate-glucuronosyltransferase 1A1 (UGT1A1) and cytochrome P450 (CYP3A) enzymes, which are responsible for DTG metabolism, resulting in reduced concentrations of DTG when co-administered with rifapentine. Decreased DTG concentrations were observed in PLWH with undetectable VLs who received 3HP together with TLD.^7^ Despite reduced DTG concentrations, VL < 50 copies/mL was maintained in all individuals through 24 weeks.^7^ However, another study in ART-naïve individuals reported a higher risk of viraemia > 50 copies/mL at 6 months in those receiving TLD and 3HP together, compared to those receiving TLD alone,^6^ consistent with outcomes in this individual who failed to achieve viral suppression within 6 months, and suggests the possible facilitation of DTG resistance among individuals receiving the concomitant therapies. Interactions between rifapentine and DTG are a growing concern in view of the expanded use of both DTG-based ART and 3HP. In this case, daily isoniazid would have been the preferred choice for TB preventive therapy, highlighting the importance of integrating HIV-TB care, and of continuous training on the implementation of 3HP in HIV programmes.

Genotypic resistance testing at the time of viraemia allowed for identification of the early emergence of DTG resistance and facilitated the choice of a PI-based regimen with which viral suppression was achieved. Adherence is difficult to confirm in routine care settings where drug-level monitoring is not readily available. Although the trial added the benefit of objectively confirming adherence, the urine tenofovir assay is limited in that it can only detect recent tenofovir use within a 7-day period and it was performed only monthly. This case highlights the benefit of early resistance testing in specific cases where risk factors such as a high pre-DTG VL or drug-drug interactions are identified in individuals with sustained viraemia on DTG-based ART.
